# Ten simple rules for collaboratively writing a multi-authored paper

**DOI:** 10.1371/journal.pcbi.1006508

**Published:** 2018-11-15

**Authors:** Marieke A. Frassl, David P. Hamilton, Blaize A. Denfeld, Elvira de Eyto, Stephanie E. Hampton, Philipp S. Keller, Sapna Sharma, Abigail S. L. Lewis, Gesa A. Weyhenmeyer, Catherine M. O’Reilly, Mary E. Lofton, Núria Catalán

**Affiliations:** 1 Australian Rivers Institute, Griffith University, Nathan, Queensland, Australia; 2 Department of Ecology and Environmental Science, Umeå University, Umeå, Sweden; 3 Marine Institute, Furnace, Newport, Co. Mayo, Ireland; 4 Center for Environmental Research, Education and Outreach, Washington State University, Pullman, Washington, United States of America; 5 UFZ, Helmholtz Centre for Environmental Research, Department of Lake Research, Magdeburg, Germany; 6 Department of Biology, York University, Toronto, Ontario, Canada; 7 Department of Biology, Pomona College, Claremont, California, United States of America; 8 Department of Ecology and Genetics/Limnology, Uppsala University, Uppsala, Sweden; 9 Department of Geography, Geology, and the Environment, Illinois State University, Normal, Illinois, United States of America; 10 Department of Biological Sciences, Virginia Tech, Blacksburg, Virginia, United States of America; 11 Catalan Institute for Water Research (ICRA), Girona, Spain; Whitehead Institute, UNITED STATES

## Introduction

Science is increasingly done in large teams [1], making it more likely that papers will be written by several authors from different institutes, disciplines, and cultural backgrounds. A small number of “Ten simple rules” papers have been written on collaboration [2, 3] and on writing [4, 5] but not on combining the two. Collaborative writing with multiple authors has additional challenges, including varied levels of engagement of coauthors, provision of fair credit through authorship or acknowledgements, acceptance of a diversity of work styles, and the need for clear communication. Miscommunication, a lack of leadership, and inappropriate tools or writing approaches can lead to frustration, delay of publication, or even the termination of a project.

To provide insight into collaborative writing, we use our experience from the Global Lake Ecological Observatory Network (GLEON) [6] to frame 10 simple rules for collaboratively writing a multi-authored paper. We consider a collaborative multi-authored paper to have three or more people from at least two different institutions. A multi-authored paper can be a result of a single discrete research project or the outcome of a larger research program that includes other papers based on common data or methods. The writing of a multi-authored paper is embedded within a broader context of planning and collaboration among team members. Our recommended rules include elements of both the planning and writing of a paper, and they can be iterative, although we have listed them in numerical order. It will help to revisit the rules frequently throughout the writing process. With the 10 rules outlined below, we aim to provide a foundation for writing multi-authored papers and conducting exciting and influential science.

## Rule 1: Build your writing team wisely

The writing team is formed at the beginning of the writing process. This can happen at different stages of a research project. Your writing team should be built upon the expertise and interest of your coauthors. A good way to start is to review the initial goal of the research project and to gather everyone’s expectations for the paper, allowing all team members to decide whether they want to be involved in the writing. This step is normally initiated by the research project leader(s). When appointing the writing team, ensure that the team has the collective expertise required to write the paper and stay open to bringing in new people if required. If you need to add a coauthor at a later stage, discuss this first with the team (Rule 8) and be clear as to how the person can contribute to the paper and qualify as a coauthor (Rules 4 and 10). When in doubt about selecting coauthors, in general we suggest to opt for being inclusive. A shared list with contact information and the contribution of all active coauthors is useful for keeping track of who is involved throughout the writing process.

In order to share the workload and increase the involvement of all coauthors during the writing process, you can distribute specific roles within the team (e.g., a team leader and a facilitator [see Rule 2] and a note taker [see Rule 8]).

## Rule 2: If you take the lead, provide leadership

Leadership is critical for a multi-authored paper to be written in a timely and satisfactory manner. This is especially true for large, joint projects. The leader of the writing process and first author typically are the same person, but they don’t have to be. The leader is the contact person for the group, keeps the writing moving forward, and generally should manage the writing process through to publication. It is key that the leader provides strong communication and feedback and acknowledges contributions from the group. The leader should incorporate flexibility with respect to timelines and group decisions. For different leadership styles, refer to [7, 8].

When developing collaborative multi-authored papers, the leader should allow time for all voices to be heard. In general, we recommend leading multi-authored papers through consensus building and not hierarchically because the manuscript should represent the views of all authors (Rule 9). At the same time, the leader needs to be able to make difficult decisions about manuscript structure, content, and author contributions by maintaining oversight of the project as a whole.

Finally, a good leader must know when to delegate tasks and share the workload, e.g., by delegating facilitators for a meeting or assigning responsibilities and subleaders for sections of a manuscript. At times, this may include recognizing that something has changed, e.g., a change in work commitments by a coauthor or a shift in the paper’s focus. In such a case, it may be timely for someone else to step in as leader and possibly also as first author, while the previous leader’s work is acknowledged in the manuscript or as a coauthor (Rule 4).

## Rule 3: Create a data management plan

If not already implemented at the start of the research project, we recommend that you implement a data management plan (DMP) that is circulated at an early stage of the writing process and agreed upon by all coauthors (see also [9] and https://dmptool.org/; https://dmponline.dcc.ac.uk/). The DMP should outline how project data will be shared, versioned, stored, and curated and also details of who within the team will have access to the (raw) data during and post publication.

Multi-authored papers often use and/or produce large datasets originating from a variety of sources or data contributors. Each of these sources may have different demands about how data and code are used and shared during analysis and writing and after publication. Previous articles published in the “Ten simple rules” series provide guidance on the ethics of big-data research [10], how to enable multi-site collaborations through open data sharing [3], how to store data [11], and how to curate data [12]. As many journals now require datasets to be shared through an open access platform as a prerequisite to paper publication, the DMP should include detail on how this will be achieved and what data (including metadata) will be included in the final dataset.

Your DMP should not be a complicated, detailed document and can often be summarized in a couple of paragraphs. Once your DMP is finalized, all data providers and coauthors should confirm that they agree with the plan and that their institutional and/or funding agency obligations are met. It is our experience within GLEON that these obligations vary widely across the research community, particularly at an intercontinental scale.

## Rule 4: Jointly decide on authorship guidelines

Defining authorship and author order are longstanding issues in science [13]. In order to avoid conflict, you should be clear early on in the research project what level of participation is required for authorship. You can do this by creating a set of guidelines to define the contributions and tasks worthy of authorship. For an authorship policy template, see [14] and check your institute’s and the journal’s authorship guidelines. For example, generating ideas, funding acquisition, data collection or provision, analyses, drafting figures and tables, and writing sections of text are discrete tasks that can constitute contributions for authorship (see, e.g., the CRediT system: http://docs.casrai.org/CRediT [15]). All authors are expected to participate in multiple tasks, in addition to editing and approving the final document. It is debated whether merely providing data does qualify for coauthorship. If data provision is not felt to be grounds for coauthorship, you should acknowledge the data provider in the Acknowledgments [16].

Your authorship guidelines can also increase transparency and help to clarify author order. If coauthors have contributed to the paper at different levels, task-tracking and indicating author activity on various tasks can help establish author order, with the person who contributed most in the front. Other options include groupings based on level of activity [17] or having the core group in the front and all other authors listed alphabetically. If every coauthor contributed equally, you can use alphabetical order [18] or randomly assigned order [19]. Joint first authorship should be considered when appropriate. We encourage you to make a statement about author order (e.g., [19]) and to generate authorship attribution statements; many journals will include these as part of the Acknowledgments if a separate statement is not formally required. For those who do not meet expectations for authorship, an alternative to authorship is to list contributors in the Acknowledgments [15]. Be aware of coauthors’ expectations and disciplinary, cultural, and other norms in what constitutes author order. For example, in some disciplines, the last author is used to indicate the academic advisor or team leader. We recommend revisiting definitions of authorship and author order frequently because roles and responsibilities may change during the writing process.

## Rule 5: Decide on a writing strategy

**Fig 1:**
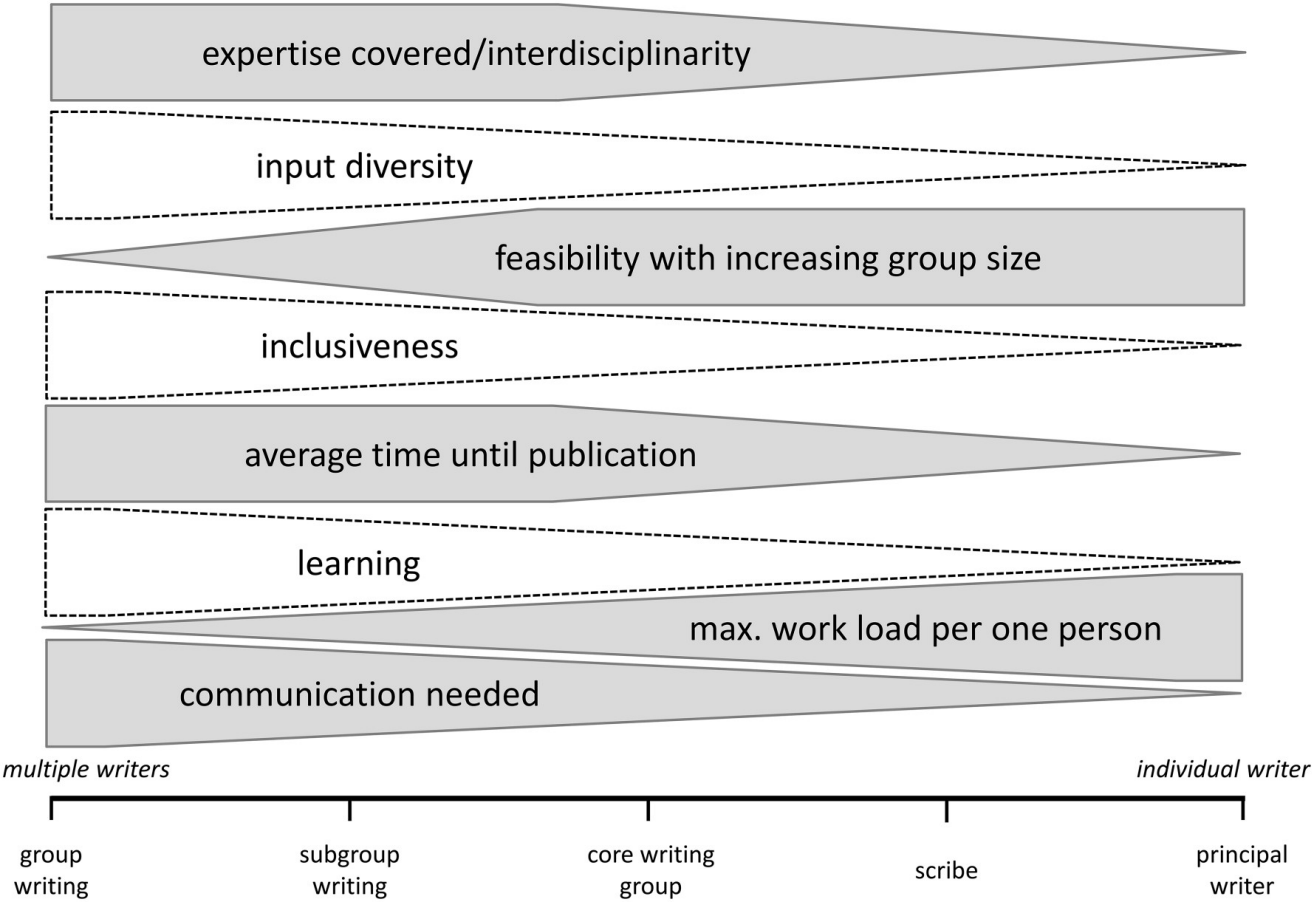
Decision chart for writing strategy. Different writing strategies ranging from very inclusive to minimally inclusive: group writing = everyone writes on everything; subgroup writing = document is split up into expertise areas, each individual contributes to a subsection; core writing group = a subgroup of a few coauthors writes the paper; scribe writing = one person writes based on previous group discussions; principal writer = one person drafts and writes the paper (writing styles adapted from [20]). Which writing strategy you choose depends on external factors (filled, gray shapes), such as the interdisciplinarity of the study or the time pressure of the paper to be published, and affects the payback (dashed, white shapes). An increasing height of the shape indicates an increasing quantity of the decision criteria, such as the interdisciplinarity, diversity, feasibility, etc.

The writing strategy should be adapted according to the needs of the team (white shapes in Fig 1) and based on the framework given through external factors (gray shapes in Fig 1). For example, a research paper that uses wide-ranging data might have several coauthors but one principal writer (e.g., a PhD candidate) who was conducting the analysis, whereas a comment or review in a specific research field might be written jointly by all coauthors based on parallel discussion. In most cases, the approach that everyone writes on everything is not possible and is very inefficient. Most commonly, the paper is split into sub-sections based on what aspects of the research the coauthors have been responsible for or based on expertise and interest of the coauthors. Regardless of which writing strategy you choose, the importance of engaging all team members in defining the narrative, format, and structure of the paper cannot be overstated; this will preempt having to rewrite or delete sections later.

For an efficient writing process, try to use the active voice in suggestions and make direct edits rather than simply stating that a section needs revision. For all writing strategies, the lead author(s) has to ensure that the completed text is cohesive.

## Rule 6: Choose digital tools to suit your needs

A suitable technology for writing your multi-authored paper depends upon your chosen writing approach (Rule 5). For projects in which the whole group writes together, synchronous technologies such as Google Docs or Overleaf work well by allowing for interactive writing that facilitates version control (see also [21]). In contrast, papers written sequentially, in parallel by subsections, or by only one author may allow for using conventional programs such as Microsoft Word or LibreOffice. In any case, you should create a plan early on for version control, comments, and tracking changes. Regularly mark the version of the document, e.g., by including the current date in the file name. When working offline and distributing the document, add initials in the file name to indicate the progress and most recent editor.

High-quality communication is important for efficient discussion on the paper’s content. When picking a virtual meeting technology, consider the number of participants permitted in a single group call, ability to record the meeting, audio and visual quality, and the need for additional features such as screencasting or real-time notes. Especially for large groups, it can be helpful for people who are not currently speaking to mute their microphones (blocking background noise), to use the video for nonverbal communication (e.g., to show approval or rejection and to help nonnative speakers), or to switch off the video when internet speeds are slow. More guidelines for effective virtual meetings are available in Hampton and colleagues [22].

In between virtual meetings, virtual technologies can help to streamline communication (e.g., https://slack.com) and can facilitate the writing process through shared to-do lists and task boards including calendar features (e.g., http://trello.com).

With all technologies, accessibility, ease of use, and cost are important decision criteria. Note that some coauthors will be very comfortable with new technologies, whereas others may not be. Both should be ready to compromise in order to be as efficient and inclusive as possible. Basic training in unfamiliar technologies will likely pay off in the long term.

## Rule 7: Set clear timelines and adhere to them

As for the overall research project, setting realistic and effective deadlines maintains the group’s momentum and facilitates on-schedule paper completion [23]. Before deciding to become a coauthor, consider your own time commitments. As a coauthor, commit to set deadlines, recognize the importance of meeting them, and notify the group early on if you realize that you will not be able to meet a deadline or attend a meeting. Building consensus around deadlines will ensure that internally imposed deadlines are reasonably timed [23] and will increase the likelihood that they are met. Keeping to deadlines and staying on task require developing a positive culture of encouragement within the team [14]. You should respect people’s time by being punctual for meetings, sending out drafts and the meeting agenda on schedule, and ending meetings on time.

To develop a timeline, we recommend starting by defining the “final” deadline. Occasionally, this date will be set “externally” (e.g., by an editorial request), but in most cases, you can set an internal consensus deadline. Thereafter, define intermediate milestones with clearly defined tasks and the time required to fulfill them. Look for and prioritize strategies that allow multiple tasks to be completed simultaneously because this allows for a more efficient timeline. Keep in mind that “however long you give yourself to complete a task is how long it will take” [24] and that group scheduling will vary depending on the selected writing strategy (Rule 5). Generally, collaborative manuscripts need more draft and revision rounds than a “solo” article.

## Rule 8: Be transparent throughout the process

This rule is important for the overall research project but becomes especially important when it comes to publishing and coauthorship. Being as open as possible about deadlines (Rule 7) and expectations (including authorship, Rule 4) helps to avoid misunderstandings and conflict. Be clear about the consequences if someone does not follow the group’s rules but also be open to rediscuss rules if needed. Potential consequences of not following the group’s rules include a change in author order or removing authorship. It should also be clear that a coauthor’s edits might not be included in the final text if s/he does not contribute on time. Bad experience from past collaboration can lead to exclusion from further research projects.

As for collaboration [2], communication is key. During meetings, decide on a note taker who keeps track of the group’s discussions and decisions in meeting notes. This will help coauthors who could not attend the meeting as well as help the whole group follow up on decisions later on. Encourage everyone to provide feedback and be sincere and clear if something is not working—writing a multi-authored paper is a learning process. If you feel someone is frustrated, try to address the issue promptly within the group rather than waiting and letting the problem escalate. When resolving a conflict, it is important to actively listen and focus the conversation on how to reach a solution that benefits the group as a whole [25]. Democratic decisions can often help to resolve differing opinions.

## Rule 9: Cultivate equity, diversity, and inclusion

Multi-authored papers will likely have a team of coauthors with diverse demographics and cultural values, which usually broadens the scope of knowledge, experience, and background. While the benefit of a diverse team is clear [14], successfully integrating diversity in a collaborative team effort requires increased awareness of differences and proactive conflict management [25]. You can cultivate diversity by holding members accountable to equity, diversity, and inclusivity guidelines (e.g., https://www.ryerson.ca/edistem/).

If working across cultures, you will need to select the working language (both for verbal and written communications); this is most commonly the publication language. When team members are not native speakers in the working language, you should always speak slowly, enunciate clearly, and avoid local expressions and acronyms, as well as listen closely and ask questions if you do not understand. Besides language, be empathetic when listening to others’ opinions in order to genuinely understand your coauthors’ points of view [26].

When giving verbal or written feedback, be constructive but also be aware of how different cultures receive and react to feedback [27]. Inclusive writing and speaking provide engagement, e.g., “*we* could do that,” and acknowledge input between peers. In addition, you can create opportunities for expression of different personalities and opinions by adopting a participatory group model (e.g., [28]).

## Rule 10: Consider the ethical implications of your coauthorship

Being a coauthor is both a benefit and a responsibility: having your name on a publication implies that you have contributed substantially, that you are familiar with the content of the paper, and that you have checked the accuracy of the content as best you can. To conduct a self-assessment as to whether your contributions merit coauthorship, start by revisiting authorship guidelines for your group (Rule 4).

Be sure to verify the scientific accuracy of your contributions; e.g., if you contributed data, it is your responsibility that the data are correct, or if you performed laboratory or data analyses, it is your responsibility that the analyses are correct. If an author is accused of scientific misconduct, there are likely to be consequences for all the coauthors. Although there are currently no clear rules for coauthor responsibility [29], be aware of your responsibility and find a balance between trust and control.

One of the final steps before submission of a multi-authored paper is for all coauthors to confirm that they have contributed to the paper, agree upon the final text, and support its submission. This final confirmation, initiated by the lead author, will ensure that all coauthors have considered their role in the work and can affirm contributions. It is important that you repeat the confirmation step each time the paper is revised and resubmitted. Set deadlines for the confirmation steps and make clear that coauthorship cannot be guaranteed if confirmations are not done.

## Conclusion

When writing collaborative multi-authored papers, communication is more complex, and consensus can be more difficult to achieve. Our experience shows that structured approaches can help to promote optimal solutions and resolve problems around authorship as well as data ownership and curation. Clear structures are vital to establish a safe and positive environment that generates trust and confidence among the coauthors [14]. The latter is especially challenging when collaborating over large distances and not meeting face-to-face.

Since there is no single “right approach,” our rules can serve as a starting point that can be modified specifically to your own team and project needs. You should revisit these rules frequently and progressively adapt what works best for your team and the project.

We believe that the benefits of working in diverse groups outweigh the transaction costs of coordinating many people, resulting in greater diversity of approaches, novel scientific outputs, and ultimately better papers. If you bring curiosity, patience, and openness to team science projects and act with consideration and empathy, especially when writing, the experience will be fun, productive, and rewarding.
